# The cultural significance of wild mushrooms in San Mateo Huexoyucan, Tlaxcala, Mexico

**DOI:** 10.1186/1746-4269-10-27

**Published:** 2014-03-05

**Authors:** Luis Enrique Alonso-Aguilar, Adriana Montoya, Alejandro Kong, Arturo Estrada-Torres, Roberto Garibay-Orijel

**Affiliations:** 1 Facultad de Agrobiología, Universidad Autónoma de Tlaxcala, Km 10.5 autopista Texmelucan-Tlaxcala, C.P. 90120 Ixtacuixtla, Tlaxcala, Mexico; 2 Centro de Investigación en Ciencias Biológicas, Universidad Autónoma de Tlaxcala, Km 10.5 autopista Texmelucan-Tlaxcala, C.P. 90120 Ixtacuixtla, Tlaxcala, Mexico; 3 Instituto de Biología, Universidad Nacional Autónoma de México, Circuito Exterior s/n, Ciudad Universitaria, D.F. 04510 Coyoacán, México

**Keywords:** Ethnomycology, Ethnobiology, Fungi, Oak forests, Free listing, Traditional knowledge

## Abstract

**Background:**

We performed an ethnomycological study in a community in Tlaxcala, Central Mexico to identify the most important species of wild mushrooms growing in an oak forest, their significance criteria, and to validate the Cultural Significance Index (CSI).

**Methods:**

Thirty-three mestizo individuals were randomly selected in San Mateo Huexoyucan and were asked seven questions based on criteria established by the CSI. Among the 49 mushroom species collected in the oak forest and open areas, 20 species were mentioned most often and were analyzed in more detail. Ordination and grouping techniques were used to determine the relationship between the cultural significance of the mushroom species, according to a perceived abundance index, frequency of use index, taste score appreciation index, multifunctional food index, knowledge transmission index, and health index.

**Results:**

The mushrooms with highest CSI values were *Agaricus campestris, Ramaria* spp., *Amanita* aff. *basii, Russula* spp., *Ustilago maydis*, and *Boletus variipes*. These species were characterized by their good taste and were considered very nutritional. The species with the lowest cultural significance included *Russula mexicana*, *Lycoperdon perlatum*, and *Strobylomyces strobilaceus.* The ordination and grouping analyses identified four groups of mushrooms by their significance to the people of Huexoyucan. The most important variables that explained the grouping were the taste score appreciation index, health index, the knowledge transmission index, and the frequency of use index.

**Conclusions:**

*A.* aff. *basii* and *A. campestris* were the most significant wild mushrooms to the people of San Mateo. The diversity of the *Russula* species and the variety of *Amanita* and *Ramaria* species used by these people was outstanding. Environments outside the forest also produced useful resources. The CSI used in Oaxaca was useful for determining the cultural significance of mushrooms in SMH, Tlaxcala. This list of mushrooms can be used in conservation proposals for the *Quercus* forests in the area.

## Abstract (es)

**Resumen (Spanish):**

**Antecedentes**

Con la finalidad de conocer las especies de hongos silvestres con mayor importancia cultural, para determinar los criterios de asignación de importancia, y para validar el índice de Significancia Cultural (ISC), se realizó un estudio en una comunidad de Tlaxcala, México.

**Métodos**

Se utilizaron siete variables incluidas en el ISC, las cuales se realizaron a 33 personas mestizas seleccionadas al azar en la comunidad de San Mateo Huexoyucan. Se seleccionaron 20 especies de hongos para determinar la importancia cultural. Se utilizaron técnicas de agrupación y de ordenación para conocer la relación entre la significancia cultural de los hongos, con base en el Índice de Abundancia Percibida, el Índice de Frecuencia de Uso, el Índice de Apreciación del Sabor, el Índice de Comida Multifuncional, el Índice de Transmisión del Conocimiento y el Índice de Salud.

Los hongos que obtuvieron los valores más altos en el ISC fueron: *Agaricus campestris, Ramaria* spp., *Amanita* aff. *basii, Russula* spp., *Ustilago maydis*, and *Boletus variipes*. Son apreciados por su sabor, porque son considerados nutritivos y por ser buenos para la salud. Los análisis realizados mostraron cuatro grupos de especies con base en su importancia para las personas de Huexoyucan. Los criterios con mayor valor que se utilizan para la asignación de importancia son: el sabor, el beneficio en la salud, la transmisión del conocimiento y la frecuencia de uso.

**Conclusiones**

*A.* aff. *basii* and *A. campestris* fueron las especies de hongos más importantes para la gente de San Mateo; sin embargo, es sobresaliente la diversidad de especies de *Russula*, *Amanita* y *Ramaria*, que son utilizadas por las personas. Se observó la relevancia que tienen los ambientes fuera del bosque como productores de recursos útiles. Además, se confirmó la efectividad del ISC utilizado de manera previa en Oaxaca, para determinar la importancia Cultural en SMH, Tlaxcala. La lista de hongos obtenida puede ser de utilidad para apoyar propuestas de conservación del bosque de encino en el área estudiada.

## Background

The cultural significance (CS) of an organism is determined by its value to a specific group of individuals [1]. Several studies have measured the CS of plants by observing their use in different regions of the world. Plant CS may be useful for decision-makers [2] who want to identify which species to protect at sites threatened by human activity. Countries such as Mexico or the United States assign plant significance by plant availability, and plant species are protected according to their environment and risk classification. However, the community actually using these plants ranks their importance differently using different criteria [3]. Unfortunately, the CS of plants or other resources that have been identified by current models are not easily compared (e.g., for specific methods or analyses) [4,1,5]. In particular, few groups have studied the CS and significance criteria of mushrooms.

In Italy, the Cultural Food Significance Index, which takes seven sub-indices into account, has been used to determine the CS of various plants, including eight species of mushrooms [6]. In Mexico, the significance of wild edible mushrooms has been assessed using precise indicators, such as mushroom name, the number of uses, and knowledge of mushroom biology, ecology, and phenology [7]. Montoya [8] measured the mushroom use value using the methodology proposed by Phillips and Gentry [9] among people of mixed Indian and Spanish descent (mestizos) living in Temezontla, Tlaxcala. However, this method is limited to the comparison of only a few mushrooms since each event must be repeated three times. Thus, the UV method does not provide enough information for understanding variables associated with CS.

Recent studies have used free listing, which considers the frequency (FM) and order (OM) of mention [10-12], as indicators of mushroom significance. This technique facilitates comparisons between communities with different ethnic origins that collect mushrooms from the same forests. FM is associated with the more expensive and less abundant mushrooms [10]. In addition, the most important mushrooms differ by community depending on the community’s cultural characteristics as well as the microenvironments where the mushrooms grow [12].

In Ixtlán de Juárez, Oaxaca, Mexico, mushroom CS has been calculated using three techniques: (1) free listing; (2) a Compound Index of CS; and (3) two questions for intracultural calculations [13]. In this study, the authors showed that knowledge distribution was not homogeneous and significantly differed by occupation and age but not gender. The importance of different mushroom species varied by sub-index, which suggested that people value different species for several different reasons. The mushrooms with the highest CS included *Cantharellus cibarius* s.l. and *A. caesarea* complex, which are considered important in other regions of Mexico. This suggested that this index may be useful in other localities in Mexico that have different characteristics.

Here, we used the method above [13] to obtain basic information on the CS of wild mushroom species for future regional or national comparisons. We determine the CS of wild mushrooms in San Mateo Huexoyucan (SMH), Tlaxcala, using an Index of CS (CSI). Mushroom CS was determined at local level, and was discussed at regional and national level. The criteria used to establish this significance were identified. This information will help investigations of traditional mushroom use and the impact of these practices on the abundance and conservation of wild mushrooms and associated plants and trees. In addition, this study helped identify the most useful mushroom species for people living near the *Quercus* forests in SMH. Mushrooms in these forests have a restricted distribution and need protection and careful management to be preserved. Thus, identifying the mushrooms that locals consider important could support protection of this site in the future.

## Methods

### Study area

**Figure 1:**
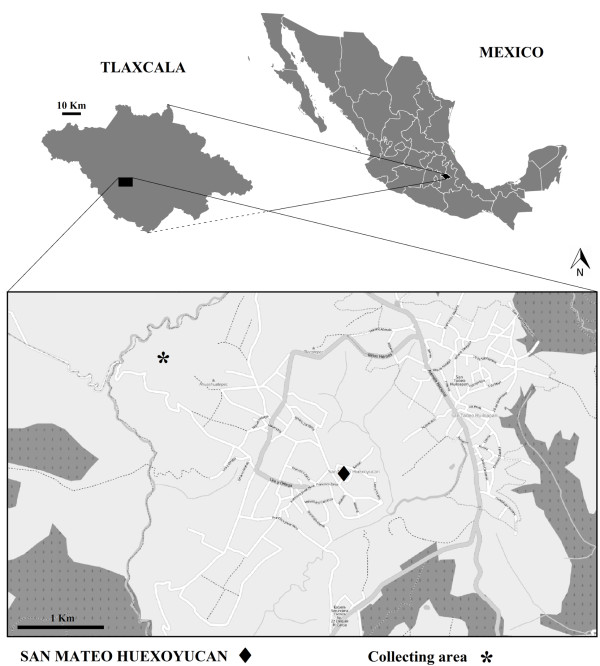
Map of the studied area of San Mateo Huexoyucan, Tlaxcala, Mexico.

SMH is located in the southwestern region of Tlaxcala, Mexico (Figure 1). This region is part of the trans-Mexican volcanic belt and has an elevation of 2,252 meters above sea level [14]. SMH belongs to the Panotla Municipality and has a total population of 1732 individuals, including 898 women and 834 men. The main activities in SMH are agriculture and stockbreeding [14]. Mushroom collection is limited to rainy seasons and is conducted in open areas surrounding the community and near oak tree forests located several kilometers away from the town. The wild vegetation observed in the upper parts of the municipality includes a community of *Pinus pseudostrobus*, *Quercus glabrescens*, *Quercus* spp., and white cedar (*Cupressus benthamii*). There is abundant secondary bush vegetation in the areas between the surrounding hills where the most common species include *Juniperus deppeana*, *Eysenhardtia polystachya*, *Opuntia spinulifera*, *Wigandia urens*, and *Amelanchier denticulata*. The most common species in the plain area of SMH include *Agave* spp., *Schinus molle*, *Tecoma stans*, *Cassia tomentosa*, *Buddleia cordata*, *Argemone* spp., *Erythrina* spp., *Ricinus communis*, *Casimiroa edulis*, *Opuntia ficus*-*indica*, *Nicotiana glauca*, and *Jacaranda mimosifolia*[15]. SMH has a sub-humid climate with summer rains and an average annual temperature of 13.6°C [16].

### Interviews (Free Listing)

Local traditional mushroom knowledge was assessed by extensive field trips and interviews with 166 people [17]. Due to the length and time involved in conducting the interviews, only 33 individuals from SMH, including 25 women and 8 men (ages 6 to 95 years) were randomly selected for in-depth interviews on the CS of local mushrooms using the CSI.

The interviews incorporated free listing where each person was asked to name 20 mushrooms to obtain mushroom FM and MO [18]. Interviewees were asked seven questions about the 20 most frequently mentioned mushroom species for a total 140 questions. The CSI was then calculated as previously described [6] and modified for mushrooms [13]. The CSI was further modified based on community-specific characteristics to exclude economic importance. Interviews were conducted between 2009 and 2011, and responses were analyzed by interviewee age and gender.

### Edible Mushroom CS Index (EMCSI)

The EMCSI of mushrooms in the SMH area was determined using six sub-indices or cultural variables and the following equation:

EMCSIspi=PAI+FUI+TSAI+MFFI+KTI+HI

where PAI is the perceived abundance index, FUI is the frequency of use index, TSAI is the taste score appreciation index, MFFI is the multi-functional food index, KTI is the knowledge transmission index, and HI is the health index.

**Figure 2:**
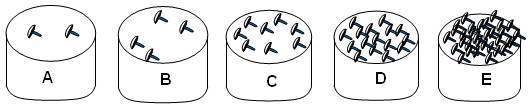
**Abundance index categories presented to the community of San Mateo Huexoyucan, Tlaxcala, Mexico. A**. Rare. **B**. Scarce. **C**. Scanty. **D**. Abundant. **E**. Very abundant.

Interviewees were asked seven questions for each mushroom species. To determine PAI, interviewees were asked “How many of these mushrooms can be found in the forest?” with an image of different abundance categories (Figure 2) [13]. The following categories were used: none or no answer (0), rare (2.5), medium (5), abundant (7.5), and very abundant (10).

**Figure 3:**
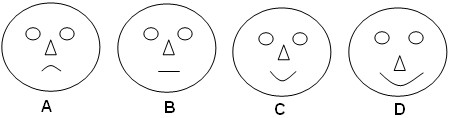
**Graphical stimulus used to calculate the taste score appreciation index (TSAI) in San Mateo Huexoyucan, Tlaxcala, Mexico. A**. I do not like. **B**. I like little. **C**. I like. **D**. I really like.

The hierarchy of answers for the other six sub-indices is shown in Table 1. Interviewees were asked, “How often do you eat this mushroom?” to determine FUI. TSAI was assessed by asking the interviewees “How much do you like this mushroom?” and “Which face expresses what you feel eating this mushroom?” while using a graphical stimulus (Figure 3) [13]. The MFFI was determined by asking interviewees “How do you cook this mushroom?” High values were allocated to species cooked alone, (i.e., dishes comprised solely of a single species of mushroom), and values diminished as other ingredients were added. The KTI assessed how many generations of SMH villagers had been picking and using mushrooms using the question “Did your mother or father, grandmother or great grandmother use this mushroom?” When interviewees reported only recently using the mushroom, they were also asked, “Who taught you the use of this mushroom?” The HI was measured by asking “In your opinion, how safe or healthy is eating this mushroom?” and “Is this mushroom harmful?”

**Table 1:** Categorization and values assigned to the answers for each Cultural Significance Sub index in San Mateo Huexoyucan, Tlaxcala, Mexico

Sub index*	**Answer**	**Value**
FUI	Never	0
	Not every year	2.5
	Every year once	5
	2-3 times a year	7.5
	4 or more a year	10
MFFI	Do not know	0
	Always mixed with other mushrooms and meat	2.5
	In a stew not as the principal element, mixed with mushrooms, without meat: with chili and mole	5
	As the principal element of a stew: in quesadillas or mushroom soup	7.5
	Cooked alone, not in stew: fried	10
KTI	Newly discovered use, cooked alone	0
	Three generations involved (he, sons and grandsons)	3.33
	Four generations involved (parents, he/she, sons and grandsons)	6.67
	Five generations involved (grandparents, parents, he/she, sons, grandsons)	10
HI	The person does not eat because of confusion with poisonous species	0
	Eaten but hurts the stomach	3.33
	Eaten with confidence and considered healthy	6.67
	Eaten because of the perceived health benefits (provides strength and is nutritious)	10
TSFAI	A	0
	B	3.33
	C	6.67
	D	10
PAI	A	0
	B	2.5
	C	5
	D	7.5
	E	10

*FUI = Frequency of Use Index, MFFI = Multifunctional Food Index, KTI = Knowledge Transmission Index, HI = Health Index, TSFAI = Test Score Flavor Appreciation Index, PAI = Perceived Abundance Index.

All sub-indices were evaluated using the same scale of 0 to 10. A value of 0 indicates no negative values. All values were given the same weight, and each sub-index was averaged across all persons interviewed.

**Figure 4:**
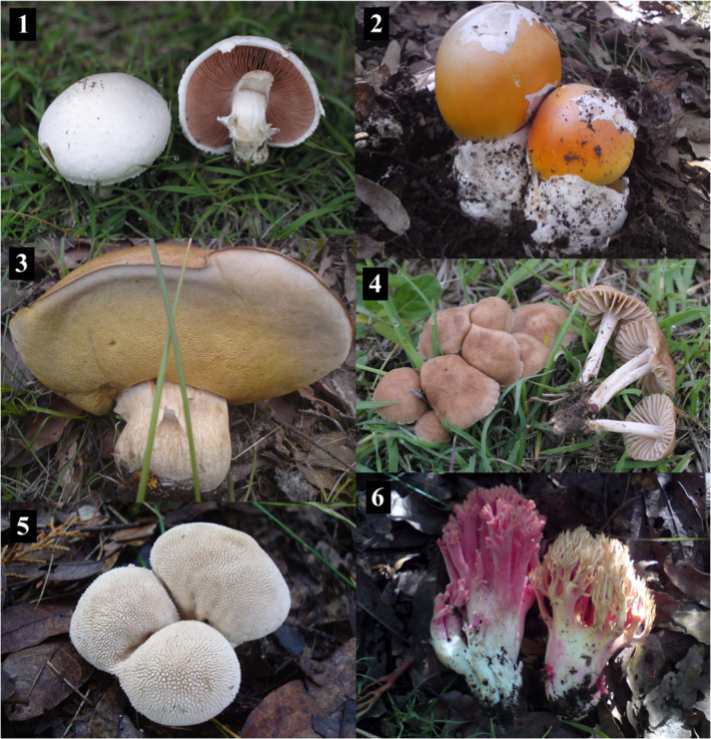
**Mushrooms photographs used as stimuli for determining the cultural significance of each species in San Mateo Huexoyucan, Tlaxcala, Mexico. 1**. *Agaricus campestris*; **2**. *Amanita* aff. *basii*; **3**. *Boletus variipes;***4**. *Marasmius oreades;***5**. *Lycoperdon perlatum;* and **6**. *Ramaria persicina.*

Photographs of fresh mushrooms were used as recognition stimuli (Figure 4). A pilot-test was conducted where the photographs were shown to several persons to evaluate their usefulness and accuracy for the different mushroom species.

The Edible Mushroom CS Pondered Index was calculated (EMCSPI) by multiplying the EMCSI by the Mention Index (MI = number of mentions/number of persons interviewed × 10).

The Ordinal Rank Value (ORV), also known as OM [19] was calculated using the following equations:

$$\mathrm{ORV}=1/\mathrm{OM}\;\mathrm{of}\;\mathrm{Sp}.1_{i};{\mathrm{ORV}}_{t}=\sum \mathrm{ORV}\;\mathrm{Sp}.1.$$

Spearman correlations were used to compare the six CS sub-indices between each mushroom species using STATVIEW software [20].

### Significance criteria

Interviewees were asked several questions to calculate the CS of a mushroom to them (intracultural evaluation) [1] and to determine their significance criteria. The following questions were asked [21]: “Which mushroom do you consider the most important?”; “Why is this mushroom important?”; “Is there any other reason why this mushroom is important to you?”; and “Anything else?” [21]. These interviews were conducted in 2010 during the dry season. The answers and their frequency were analyzed. The significance criteria mentioned most frequently were considered most important. Using these data and the results of an ethnomycological analysis previously conducted in the community [17], the Economic Index established by Garibay-Orijel *et al.*[13] could be disregarded. Mushrooms were most frequently used for eating, and only a few individuals collected mushrooms to sell in the SMH community.

### Similarity and grouping analyses

The relationships between mushroom significance calculated by the CS sub-indices were analyzed using the Index of Euclidean distance. Grouping was analyzed using a principal components analysis (PCA), where rows identified the species that formed groups and columns determined the relationship between the six CS sub-indices. A multi-dimensional scaling (MDS) analysis was also conducted to confirm and improve the PCA results. The original matrix included 20 mushroom species (those mentioned by 10% of the persons interviewed), and the values obtained for each sub-index and the pondered index. These analyses were performed using NTSYS-pc software [22].

### Mushroom collection, characterization, and identification

Mushrooms were collected from the *Quercus* forest closest to SMH during the rainy seasons of 2009 and 2010. SMH families and individuals, including seniors and/or children, accompanied the investigators during collection. Traditional mushroom names, sampling areas, and the season mushrooms were collected were also recorded. Mushrooms were collected and processed as previously described [23] and then were stored at the TLXM Herbarium of the Autonomous University of Tlaxcala. During the rainy season of 2010, the wild mushrooms were collected, photographed, and subsequently identified using general and specialized field guides according to genus. In addition, specialized literature was reviewed [24-27].

## Results and discussion

We recorded a total of 69 traditional mushroom names, which corresponded to 46 wild edible mushroom species. Several traditional names referred to the same species of mushroom (Table 2). The SMH community greatly appreciated the local mushrooms and considered these species culturally important. The people interviewed possessed a lot of traditional knowledge that was used to identify mushrooms, name mushroom harvesting time and locations, cook and preserve different mushrooms, determine which species were poisonous. The community did not only use forest mushrooms; species that grew in open areas, such as *A. campestris*, *M. oreades*, *C. cyathiformis*, and *L. perlatum*, were also considered very important. Although some individuals sold mushroom products, many species were used only for self-consumption, and both men and women of all ages collected and ate mushrooms. Many *Russula* and *Amanita* species were highly valued compared to the limited number of mushroom species used in coniferous forest areas of Tlaxcala [17].

**Table 2:** Traditional names used for wild edible mushroom species in San Mateo Huexoyucan, Tlaxcala, Mexico

**Species name**	**Traditional name**
1- *Agaricus bisporus* (J.E. Lange) Imbach	Champiñón (button mushroom)
2- *Agaricus campestris* L.	Hongo de agua (water mushroom)
3- *Amanita* aff*. basii* Guzmán & Ram.-Guill.	Hongo huevo (egg mushroom)
	Amarillo (yellow)
	Totolnanacatl
	Hongo de ladera (mountain mushroom)
	Huevo de llano (mushroom of the plains)
	Estenanacatl
4- *Amanita muscaria* (L.) Lam.	Hongo rojo de ajonjolí (red sesame mushroom)
5- *Amanita rubescens* Pers.	Venadito (little deer)
6- *Amanita* gpo. *vaginata* (Bull.) Lam.	Arriero
7- *Amanita* sp*.*	Mosca venenoso (poisonous fly)
8- *Boletus subvelutipes* Peck	Pata de toro (bull’s hoof)
	Pante morado (purple pante)
	Panza que se pone verde (greenish belly)
	Panza amarilla que no se come (inedible yellow belly inedible)
	Panza de burro (donkey’s belly)
9- *Boletus variipes* Peck	Pantenanacatl
10- *Boletus* sp.	Chipo de toro (bull’s mug)
11- *Boletus* sp*.*	Pante rosa (pink pante)
12- *Boletus* sp*.*	Panza coloradita (little red belly)
13- *Boletus* sp*.*	Porositos rojitos (little red pore)
14- *Calvatia cyathiformis* (Bosc) Morgan	Cepamil
	Cabezas (heads)
15- *Clitocybe* aff*. gibba* (Pers.) P. Kumm.	Hongo trompeta (trumpet mushroom)
	Campanita (little bell)
17- *Cantharellus* aff*. cibrius* Fr.	Tecositas
18- *Hypomyces lactifluorum* (Schwein.) Tul. & C.Tul.	Oreja de cochino (pig’s ear)
19- *Lactarius lacteolutescens* Montoya, Bandala & G. Moreno	Oyamel
20- *Lactarius indigo* (Schwein.) Fr.	Azules (blue mushrooms)
	Azul de encino (blue oak mushroom)
	Azul de ocote (blue ocote mushroom)
21- *Lactarius psammicola* A.H. Sm.	Oyamelito grande (big oyamelito)
22- *Lactarius yazooensis* Hesler & A.H. Sm.	Oyamelito
23- *Leccinum* aff*. rugosiceps* (Peck) Singer	Panzas (bellies)
	Panza blanquita (white belly)
**Species name**	**Traditional name**
24- *Lycoperdon perlatum* Pers.	Borreguitos (little lambs)
	Bolitas blancas de encino (white oak balls)
25- *Lyophyllum* aff. *decastes* (Fr.) Singer	Jicarita,
	Clavito (little clove)
26- *Marasmius oreades* (Bolton) Fr.	Xolete café (brown xolete)
	Xolete de encino (oak xolete)
	Xolete de llano (flat xolete)
27- *Ramaria* aff*. cystidiophora* (Kauffman) Corner	Escobeta amarilla (yellow broom)
28- *Ramaria botrytoides* (Peck) Corner	Escobeta (broom)
	Escobeta blanca (white broom)
29- *Ramaria persicina* Cázares	Escobeta rosa (pink broom)
	Escobeta anaranjada (orange broom)
	Escobeta roja (red broom)
30- *Ramaria* sp*.*	Escobeta gris (gray broom)
31- *Ramaria* sp*.*	Escobeta cafecita (little brown broom)
32- *Ramaria* sp*.*	Escobeta morada (purple broom)
33- *Ramaria* sp*.*	Barbas de chivo (goatee beards)
34- *Russula* aff*. anthracina* Romagn.	Hongo de chivo (goatee mushrooms)
35- *Russula cyanoxantha* (Schaeff.) Fr.	Pastelito morado (little purple little cake)
36- *Russula delica* Fr.	Blanco de ocote (ocote white ocote mushroom)
37- *Russula* aff*. macropoda* Singer	Hongo colorado (red mushroom)
	Rojos de encino (red oak red mushroom)
	Tecax rojo (red tecax)
	Chinanacatl
38- *Russula mariae* Peck	Pastelito rojito (red little cake)
39- *Russula mexicana* Burl.	Hongo rojo (red mushroom)
40- *Russula romagnesiana* Shaffer	Azteca
41- *Russula* sp*.*	Pastelitos (little cake)
42- *Russula* sp.	Pastelito azul (little blue little cake)
43- *Russula* sp*.*	Pastelito ladrillo (brike little cake)
44- *Strobilomyces strobilaceus* (Scop.) Berk.	Trompa de toro (bull’s horn)
	Chipo de toro (bull’s mug)
45- *Suillus* aff*. glandulosipes* Thiers & A.H. Sm.	Panza pardita (brown belly)
46- *Ustilago maydis* (DC.) Corda	Cuitlacoche (corn smut)

### Frequency of mention

The mushroom species mentioned most often by the 33 individuals interviewed included *A.* aff. *basii* (31 mentions), *A. campestris* (30 mentions), *Ramaria* spp. (22 mentions), *Russula* spp. (17 mentions), *Ustilago maydis* (17 mentions), *Boletus variipes* (13 mentions), and *M. oreades* (12 mentions; Table 2). Only four of these mushrooms species were mentioned by more than 50% of interviewees, followed by six species mentioned by more than 30% of interviewees and 10 species mentioned by less than 30% of interviewees. A previous study reported similar results in Ixtlán de Juárez, Oaxaca [13]. In that research, 95 individuals were interviewed, and 21 traditional mushroom names, corresponding to 37 mushroom species, were identified. *Amanita caesarea* complex, *Ramaria* spp., *Neolentinus lepideus*, and *Agaricus pampeanus* were recognized by more than 50% of those interviewed. Several of these mushrooms are known nationally or globally, while others have economic importance. Most of these mushrooms were highly prized for their taste. Other mushrooms, while mentioned less frequently, were also used and appreciated, and individuals were very familiar with these species.

#### Frequency of Use Index (FUI)

The species consumed more than once a year included *A.* aff. *basii, A. campestris,* and *Ramaria* spp. (FUI > 6.0 for all three) as well as *Russula* spp*.* (FUI = 5.3). *A.* aff*. basii* has a distinctive and peculiar taste compared to the other mushrooms collected, and the SMH community preferred this species over the others and collected and used these mushrooms the most frequently. The abundance and diversity of *Russula* mushrooms contributed to their high FUI value.

#### Multi-functional Food Index (MFFI)

Two mushroom genera and three species were preferred for cooking alone (in stock or fried and without the addition of any meat): *Ramaria* spp*.* and *Russula* spp. (MFFI > 8.0 for both) and *A. campestris, B. variipes*, and *A.* aff. *basii* (MFFI > 6.0 for all three). These taxa had the highest MFFI values. The SMH community did not typically cook *B. variipes* (MFFI = 6.6) with other mushrooms or ingredients; similarly, *A.* aff. *basii* also tended to be cooked alone. Both these species were compared with meat, and the respondents implied that these mushrooms had similar nutritional properties to chicken since the cooking stock prepared from these mushrooms exhibited the same yellow color as that from chicken. In the MFFI sub-index, *Ramaria* and *Russula* mushrooms were significant from a gastronomical point of view; these mushrooms were versatile, and different species could be cooked several ways without including other food ingredients. These high MFFI values could be attributed to the large variety of species belonging to these two genera.

#### Knowledge Transmission Index (KTI)

The ten mushroom species in the SMH community with the deepest generational roots included: *A. campestris, A.* aff*. basii, Ramaria* spp., *U. maydis,* and *Russula* spp. (KTI > 6.0 for all) as well *as B. variipes*, *M. oreades*, *H. lactifluorum*, *L. yazooensis*, *Calvatia cyathiformis*, and *P. opuntiae*. Four or five generations transmitted mushroom knowledge from one generation to the next for many generations. *A. campestris* had the highest KTI value, making these mushrooms the most significant in this sub-index. In addition, *U. maydis* ranked fourth, which suggested that this mushroom was familiar to many generations of the SMH community.

#### Health Index (HI)

The interviewees believed that all edible wild mushrooms were nutritional and healthy because they did not contain chemical substances, were 100% natural, and could be eaten with confidence. *A. campestris* (HI > 7.0) was the most significant species in this sub-index. The next most frequently eaten mushrooms with health benefits were *A.* aff*. basii, Ramaria* spp., *Russula* spp.*,* and *U. maydis* (HI > 6.0) and *B. variipes* (FUI = 4.9). Other mushrooms were considered healthy but had lower HI values (Table 1); these included *S. strobilaceus, P. opuntiae, L. indigo, C. cyathiformis, L. yazooensis, C.* aff. *gibba*, and *C.* gpo*. cibarius.* Most interviewees agreed that there were no known incidences of poisoning associated with eating forest-grown mushrooms.

#### Perceived Abundance Index (PAI)

The mushrooms considered the most abundant included *A. campestris, Russula* spp., *A.* aff*. basii*, and *Ramaria* spp. (PAI > 8.0). Mushrooms that were considered rarer were *M. oreades* (PAI = 3.6), *B. variipes* (PAI = 3.4), and *L. yazooensis* (PAI = 2.1)*.* The perceived abundance of these mushrooms reflected the actual abundance of the mushrooms. Collectors walked long distances to obtain these mushrooms and frequently moved through different types of vegetation at varying altitudes. Their experiences gave the community a clear idea of the abundance of various mushrooms.

Ethnomycological [10] and ecological [28] studies have been conducted in a forest near the SMH that also contains *Quercus* trees. These studies found that the same species present in that forest were almost identical to those in the SMH forest in this study. Only a few *A.* aff. *basii* were found in the other forest, which was located near the San Francisco Temezontla community; this species was also rarely detected in SMH. Thus, *A.* aff. *basii* are an unusual, rare, and highly prized species. Additional ecological studies, particularly of SMH, are needed to determine the actual abundance of *A.* aff. *basii* and to corroborate its perceived abundance within the community.

High abundance and variety values were observed for the diverse *Russula* species, which was consistent with the perceptions of the SMH community. The significance attributed to *Russula* mushrooms is also consistent with values obtained by the MFFI. These findings agree with the perceived abundance of *Russula* mushrooms in Ixtlán de Juárez, Oaxaca [13].

#### Taste Score Appreciation Index (TSAI)

The species with the best TSAI values included: *A.* aff. *basii, A. campestris, Ramaria* spp.*,* and *Russula* spp. (TSAI > 8.0 for all); *U. maydis*, *B. variipes*, *M. oreades*, and *H. lactifluorum* (TSAI > 4.0 for all); *L. indigo*, *P. opuntiae*, and *L. yazooensis* (TSAI > 3.0 for all three); *C. cyathiformis* (TSAI >2.9); and *C. aff. gibba, A.* aff*. rubescens*, and *C.* gpo*. cibarius* (TSAI < 2.0 for all three)*.* Mushrooms with reportedly simpler tastes were *R. mexicana*, *L. perlatum, L.* gpo*. decastes, A.* gpo. *vaginata*, and *S. strobilaceus*. Interviewees did not consider any of the mushrooms to have an unpleasant taste. This is consistent with the observed significance criteria for mushrooms in the SMH community, indicating that taste is the primary criterion for determining mushroom preference.

#### Edible Mushrooms CS Index (EMCSI)

The EMCSI values ranged from 2.3 for *S. strobilaceus* to 41.9 for *A. campestris* (Table 3). Mushrooms with the most CS included *A. campestris, Ramaria* spp., *Amanita* aff. *basii, Russula* spp., *U. maydis*, and *B. variipes*, which are all valued for taste and nutritional value. The species with the least CS included *R. mexicana*, *L. perlatum*, and *S. strobilaceus*.

**Table 3:** Cultural significance values for edible wild fungi in San Mateo Huexoyucan, Tlaxcala, Mexico

**Species**	**M**	**ORV**	**MI**	**FUI**	**MFFI**	**KTI**	**HI**	**PAI**	**TSAI**	**EMCSI**	**EMCSPIm**	**IntraC**
*Amanita* aff. *basii*	31	12.912	9.394	7.720	6.591	6.667	6.770	4.621	8.939	41.309	388.052	14
*Agaricus campestris*	30	20.077	9.091	6.818	6.970	6.768	7.174	5.606	8.587	41.923	381.117	12
*Ramaria* spp.	22	3.710	6.667	6.742	8.788	6.465	6.669	4.621	8.384	41.670	277.800	
*Russula* spp*.*	17	3.360	5.152	5.303	8.561	6.162	6.467	5.000	7.375	38.868	200.228	
*Boletus varipes*	13	3.558	3.939	3.485	6.667	5.152	4.951	3.409	5.355	29.019	114.318	1
*Marasmius oreades*	12	7.376	3.636	3.333	5.909	4.546	4.648	3.636	4.345	26.418	96.065	1
*Ustilago maydis*	9	2.116	2.727	2.576	6.212	6.263	6.467	1.061	6.971	29.550	80.591	
*Hypomices lactifluorum*	11	1.743	3.333	2.045	2.500	3.738	3.436	1.818	4.041	17.578	58.595	
*Calvatia cyathiformis*	12	7.171	3.636	1.970	3.030	3.233	2.628	1.970	2.930	15.760	57.308	3
*Lactarius indigo*	12	5.411	3.636	2.121	2.727	2.526	2.930	1.818	3.435	15.557	56.571	2
*Pleurotus opuntiae*	11	1.912	3.333	1.742	3.182	2.930	3.334	0.833	3.233	15.255	50.848	
*Lactarius yazooensis*	9	4.476	2.727	1.591	2.879	2.829	2.627	2.197	3.031	15.154	41.328	
*Clitocybe* aff. *gibba*	9	1.296	2.727	0.758	1.515	1.313	1.212	0.758	1.212	6.768	18.460	
*Amanita* aff*. rubescens*	8	1.454	2.424	0.758	1.212	0.808	0.808	0.455	1.010	5.051	12.245	
*Cantharellus* aff. *cibarius*	7	0.934	2.121	0.606	0.758	1.212	1.212	0.682	1.010	5.481	11.626	
*Russula mexicana*	8	2.710	2.424	0.530	0.758	0.606	0.606	0.530	0.707	3.738	9.062	
*Lycoperdon perlatum*	7	1.990	2.121	0.227	0.682	0.707	0.606	0.303	0.707	3.233	6.858	
*Lyophyllum* gpo. *decastes*	6	1.054	1.818	0.303	0.909	0.606	0.606	0.303	0.707	3.435	6.245	
*Amanita* gpo*. vaginata*	5	1.768	1.515	0.379	0.758	0.808	0.707	0.455	0.606	3.713	5.625	
*Strobilomyces strobilaceus*	3	0.535	0.909	0.303	0.455	0.404	0.505	0.227	0.505	2.399	2.181	

M: Number of mentions in free listings, ORV: Ordinal Rank Value, MI: Mention Index, FUI: Frequency of Use Index, MFFI: Multifunctional Food Index, KTI: Knowledge Transmission Index, HI: Index of Health, PAI: Perceived Abundance Index, TSAI: Taste Score Appreciation Index, EMCSI: Index of Cultural Significance for Wild Edible Mushrooms, EMCSPIm: Edible Mushroom Cultural Significance Pondered, IntrC: Mention Number in the intracultural evaluation.

*A. campestris* was considered the most significant mushroom, primarily because of its taste (8.59), nutritional value (7.1), and cooking versatility (6.9). *A. campestris* had higher values in four of the sub-indices than *A*. aff. *basii*, which has reduced availability at the start of the rainy season. The TSAI, HI, KTI, and FUI sub-indices contributed the most to mushroom significance. In Ixtlán de Juárez, Oaxaca, the mushroom species analyzed produced different values in each sub-index. Combined with results from the present study, these data indicate that people value each species of mushroom for different reasons.

### Edible Mushrooms CS Pondered Index (EMCSPI)

The EMCSPI represents the significance of a mushroom species to the SMH community after the number of persons mentioning each mushroom and the total number of persons (N = 33) interviewed are taken into account. EMCSPI values ranged from 2.18 to 388.05. Using this measure, the species with the most CS included *A.* aff*. basii* (388.05)*, A. campestris* (381.17)*, Ramaria* spp.*, Russula* spp. (277.80), and *B. variipes* (114.32)*.* These species, except for a single *Boletus* species, are consistent with those observed in previous studies. Here, the ranking of these species’ significance changed because the value obtained for the mention index (MI) was included. Thus, the EMCSPI is based on the relative value of the number of mentions, and species with many mentions have a higher value. The remaining mushrooms had values under 100; the species with the least CS were *R. mexicana*, *L. perlatum, L.* gpo*. decastes, A.* gpo*. vaginata*, and *S. strobilaceus*.

#### Mention Index (MI)

The MI values for each mushroom species are shown in Table 1. The two species with the highest MI values were the same as those considered most important by the FUI, KTI, and TSAI sub-indices. However, these results conflict with ORVs; more interviewees mentioned *A*. aff. *basii* but did not consider it to be the most significant.

#### Ordinal Rank Value (ORV)

*A. campestris* had the highest ORV (20.0), which was consistent with KTI, HI, and PAI values. These results indicate that more individuals gave this species the most significance, followed by *A.* aff. *basii* (12.9). Both species had the most significance based on MI (Mention Index) and ORV; thus, these mushrooms are the most important species to the SMH community (Table 3). *M. oreades* (7.3), *C. cyathiformis* (7.1), and *L. indigo* (5.4) were not mentioned as frequently when compared to the *A. campestris* and *A.* aff. *basii* but still ranked among the top five most significant species. Thus, this sub-index is a reliable indicator of CS, although the results were not consistent with the MI.

Correlation analyses showed that all sub-indices were significantly correlated. Similar results were obtained for all cases (r_s_ = 0.54-0.99, P < 0.0001). This confirms that all sub-indices contributed in the same way to determining the CS of the different mushroom species.

### Significance criteria

In the present study, six mushroom species were considered significant by intracultural evaluation: *A.* aff*. basii* (14 people), *A. campestris* (12), *C. cyathiformis* (3), *L. indigo* (2), *B. variipes* (1), and *M. oreades* (1). The interviewees reported 11 reasons for the significance of these mushrooms: taste (27 mentions [m]), naturally grown (16 m), nutritional value (14 m), lack chemical additives (9 m), abundance in the forest (8 m), meat-like taste (3 m), rapid growth (2 m), pleasant smell (2 m), frequent consumption (2 m), expensive price (1 m), and non-poisonous (1 m). These results are consistent with the CS obtained using the various indices (EMCSI and EMCSPI).

### Similarity analysis

**Figure 5:**
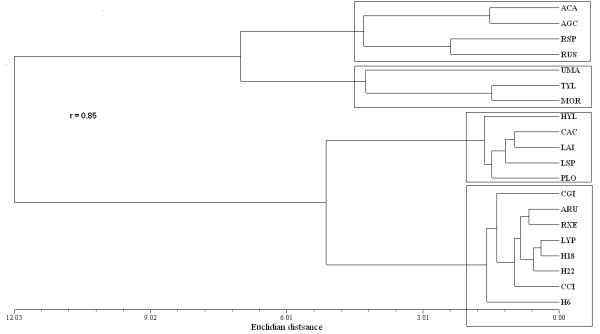
**Similarities between wild edible mushroom species according to their cultural significance in San Mateo Huexoyucan, Tlaxcala, Mexico.** The rectangles show major mushroom groups. ACA: *Amanita* aff. *basii*; AGC: *Agaricus campestris*; RSP: *Ramaria* spp.; RUS: *Russula* spp.; UMA: *Ustilago maydis*; TYL: *Boletus variipes*; MOR: *Marasmius oreades*; HYL: *Hypomyces lactifluorum*; CAC: *Calvatia cyathiformis*; LAI: *Lactarius indigo*; LSP: *Lactarius yazooensis*; PLO: *Pleurotus opuntiae*; CGY: *Clitocybe* aff. *gibba*; ARU: *Amanita rubescens*; RXE: *Russula mexicana*; LYP: *Lycoperdon perlatum*; H18: *Lyophyllum* aff. *decastes*; H22: *Amanita* gpo. *vaginata*; CCI: *Cantharellus* gpo. *cibarius*; and H6: *Srobilomyces strobilaceus*.

The phenogram in Figure 5 shows two groups of species (Euclidian distance of 12.03). Group A is comprised of the species with the most CS based on the diverse variables included in the analysis (*A.* aff*. basii*, *A. campestris*, *Ramaria* spp., *Russula* spp., *U. maydis, B. variipes*, and *M. oreades*; Table 3). The species most similar in this group are *B. variipes* and *M. oreades*, followed by *A.* aff. *basii* and *A. campestris.* Group B is divided into two sub-groups (Euclidian distance of 5): (a) *H. lactifluorum*, *C. cyathiformis*, *L. indigo*, *Lactarius* sp., and *P. opuntiae*; and (b) *C. gibba*, *A. rubescens*, *R. mexicana*, *L. perlatum, L.* aff. *decastes*, *Amanita* gpo. *vaginata*, *C.* gpo. *cibarius*, and *S. strobilaceus*. In the first Group B sub-group, the closest species are *C. cyathyformis* and *L. indigo*. In the latter sub-group, *S. strobilaceus* is independent from all other members with a Euclidian distance of 2, while *L. perlatum* and *L.* gpo*. decastes* are most similar with identical values in four sub-indices (HI, PAI, TSAI, and EMCSI).

### Multivariate analyses

**Figure 6:**
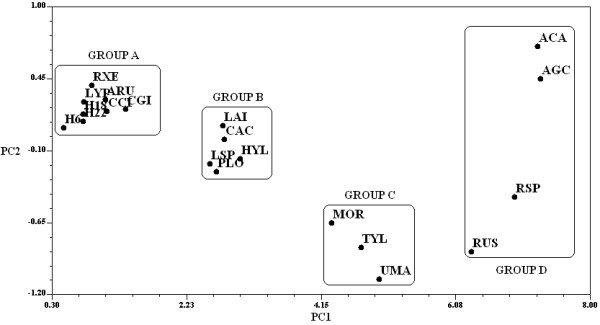
**Similarities between wild edible mushrooms using the principal component analysis and the cultural significance values obtained in San Mateo Huexoyucan, Tlaxcala, Mexico.** The inside rectangles divide major groups of mushrooms; PC: Principal Component; ACA: *Amanita* aff. *basii*; AGC: *Agaricus campestris*; RSP: *Ramaria* spp.; RUS: *Russula* spp.; UMA: *Ustilago maydis*; TYL: *Boletus variipes*; MOR: *Marasmius oreades*; HYL: *Hypomyces lactifluorum*; CAC: *Calvatia cyathiformis*; LAI: *Lactarius indigo*; LSP: *Lactarius yazooensis*; PLO: *Pleurotus opuntiae*; CGY: *Clitocybe* aff. *gibba*; ARU: *Amanita rubescens*; RXE: *Russula mexicana*; LYP: *Lycoperdon perlatum*; H18: *Lyophyllum* aff. *decastes*; H22: *Amanita* gpo. *vaginata*; CCI: *Cantharellus* gpo. *cibarius*; and H6: *Srobilomyces strobilaceus*.

A PCA was used for multivariate analyses (Figure 6). Comparisons between principal component 1 (PC 1) vs. PC 2 resulted in four main groups of mushrooms. Group A had values <2.23 (Figure 6, left), Group B had values >2.23, and Groups C and D had values >4.15 (Figure 6, right). Group A is comprised of the species with the least significance: *R. mexicana*, *A. rubescens*, *L. perlatum, L.* aff. *decastes*, *C.* gpo*. cibarius*, *C. gibba*, *A.* gpo. *vaginata*, and *S. strobilaceus*. Group B is comprised of five species with more significance than those in group A: *L. indigo, C. cyathiformis, H. lactifluorum*, *L. yazooensis*, and *P. opuntiae*. Group C is comprised of *M. oreades*, *B. variipes* and *U. maydis*, and Group D is comprised of *Russula* spp., *Ramaria* spp., *A. campestris*, and *A.* aff. *basii. A. campestris* and *A.* aff. *Basii* were the most distant in Group D. Similarity between these two genera were observed among the mushrooms in this group, which included several species mentioned by more than half the interviewees (*Russula* spp. and *Ramaria* spp).

**Figure 7:**
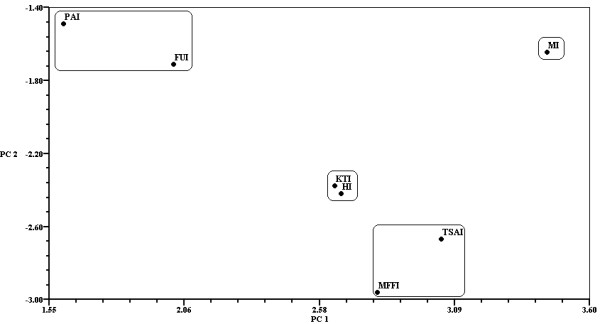
**Principal component analysis results after comparing the sub-indices used to determine the cultural significance of wild mushrooms in San Mateo Huexoyucan, Tlaxcala, Mexico.** PC: Principal Component; FUI: Frequency of Use Index; MI: Mention Index; KTI: Knowledge Transmission Index; HI: Health Index; TSAI: Taste Score Appreciation Index; PAI: Perceived Abundance Index; MFFI: Multifunctional Food Index.

The different species were grouped based on similar values obtained in the different sub-indices. The first three components explained 99.24% of the variation using Eigen analyses. The most important variables that affected grouping were the TSAI (0.99), HI (0.98), KTI, and FUI (0.97) in PC 1; the MI (0.31) and MFFI (-0.23) in PC 2; and the PAI (0.32) in PC 3. These species were hierarchically arranged based on their significance to the SMH community, which was reflected in the sub-indices values. The PCA, which compared the sub-indices, showed that the PAI and FUI grouped together while the HI and KTI, the TSAI and MFFI, and the MI did not (Figure 7).

Six mushroom species explained the observed variation in PC 1 (H6, *C. cyathiformis*, *L. indigo*, *C. gibba*, H18, and *L. perlatum*; values >0.90) and PC 2 (*Russula* species and *B. variipes*; values > 0.90; Table 3). The first three components explained 92.96% of the variation. These results are consistent with those obtained from the sub-indices. *A. campestris* was the most significant mushroom species due to its abundance (PAI), nutritional value (HI), and generational knowledge (KTI); similarly, *A.* aff. *basii* was also significant due to its frequent use (FUI), taste (TSAI), and reputation (MI). High values were also obtained for the *Russula* and *Ramaria* mushrooms since these species were considered sufficiently nutritional and were consumed alone.

**Figure 8:**
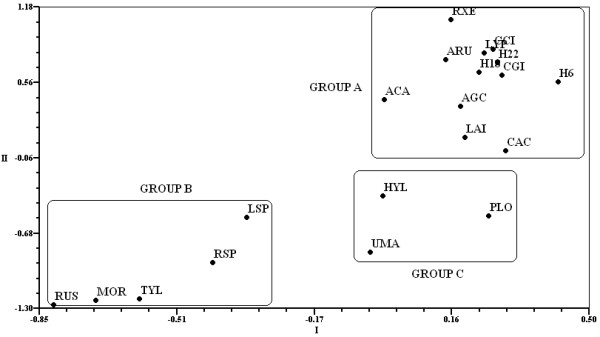
**Similarities between wild edible mushrooms according to the multidimensional scaling analysis and CS values obtained in San Mateo Huexoyucan, Tlaxcala, Mexico. I = Dimension I, II = Dimension II.** The inside rectangles divide major groups of mushrooms. ACA: *Amanita* aff. *basii*; AGC: *Agaricus campestris*; RSP: *Ramaria* spp.; RUS: *Russula* spp.; UMA: *Ustilago maydis*; TYL: *Boletus variipes*; MOR: *Marasmius oreades*; HYL: *Hypomyces lactifluorum*; CAC: *Calvatia cyathiformis*; LAI: *Lactarius indigo*; LSP: *Lactarius yazooensis*; PLO: *Pleurotus ountiae*; CGY: *Clitocybe* aff. *gibba*; ARU: *Amanita rubescens*; RXE: *Russula mexicana*; LYP: *Lycoperdon perlatum*; H18: *Lyophyllum* aff. *decastes*; H22: *Amanita* gpo. *vaginata*; CCI: *Cantharellus* gpo. *cibarius*; and H6: *Srobilomyces strobilaceus*.

The multidimensional scaling analysis (MDS) had a stress value of 0.2249 and had results consistent with those of the PCA for grouping some species at the maximum number of iterations (Figure 8): Group A) *R. mexicana*, *L. perlatum*, *A. rubescens*, *C. cibarius*, *C. gibba*, *L*. aff *decastes*, *A*. aff. *basii*, *A*. gpo. *vaginata*, *A. campestris*, *S. strobilaceus, L. indigo,* and *C. cyathiformis*; Group B) *Russula* spp., *Ramaria* spp. *M. oreades, Lactarius* sp*.,* and *B. variipes*; and Group C) *H. lactifluorum*, *Ustilago maydis*, and *P. opuntiae*. All sub-indices had similar contributions to the CS of the mushroom species, and no significant differences were observed. However, *A. campestris* or *A*. aff. *basii* remained the most significant, and the different groups formed by both analyses contained species with similar significance values.

The economic index used in Ixtlán was eliminated from the present study because mushrooms are primarily used for food in SMH and have little economic importance. One of the main limitations of our study was the lack of a complete list of wild mushrooms that grow in the SMH, which might have affected the calculated index values. We identified three *Ramaria* species (*R. suaveolens, R. botrytoides*, and *R. percisina*) and ten *Russula* genus species (*R. anthracina*, *R. cyanoxantha, R.* aff. *decipiens, R. delica, R. macropoda, R. mariae, R. mexicana*, *R. ornaticeps, R. romagnesiana*, and *R. xerampelina*). Other species of both genera (between four to six more species) were acknowledged and used by the local people but only had generic names and were not accurately identified*.*

The *Quercus* forests have a diverse selection of wild mushroom species, particularly mushrooms of the *Amanita*, *Ramaria*, and *Russula* genera. While free listing, individuals reported common names that were not always associated with specific taxa. Because many traditional criteria, such as odor and taste, are used to identify mushrooms with similar morphologies, using photographs as a stimulus also had severe limitations that could affect the CS calculated for some species. The high CS of *A.* aff*. basii* in the SMH community was one of the most relevant results of this study. This mushroom grows in oak forests in the center of Mexico. Several studies of mushroom CS in Mexico have shown that certain species have greater CS. *Ramaria* spp., *B. pinophilus, A.* aff. *basii*, *C.* gpo. *cibarius*, and *L. decastes* are the most frequently mentioned species in the temperate areas of Mexico [14,10,29,11,31]. *A.* gpo*. caesarea* is well known at a broad geographic level and is highly valued in Mexico, Turkey, and Nepal. In contrast, *B*. gpo. *pinophilus* is highly valued in Europe and Central America, and *C*. gpo. *cibarius* is highly valued in 45 different countries worldwide [32]. Notably, in different cultures in Mexico, certain mushroom species are particularly relevant, including: *Turbinellus floccosus* (Schwein.) Earle ex Giachini and Castellanoin in San Isidro Buensuceso, Tlaxcala [10]; *Neolentinus lepideus* in Ixtlán de Juárez, Oaxaca [11]; and *Helvella* spp. in Corral de Piedra and San Juan in the Amanalco municipality [32]. This pattern is also observed worldwide [33].

The present study also showed that mushrooms growing in open areas, such as *A. campestris*, *M. oreades*, *U. maydis*, and *C. cyathiformis*, also had CS. This suggests that the relevance of other environments where saprotrophic mushrooms are available is also important. SMH is located relatively far from the forest, and mushroom collectors have to pass through farming areas and plains to reach it. When passing through these open areas, collectors also gather any mushrooms they find in those environments. During the dry season, uncontrolled fires decimate the forests and generate many large open areas. Therefore, mushrooms outside the forest become more important as an available resource for the local population.

## Conclusions

The edible mushroom species with the most CS to the SMH community were *A.* aff. *basii* and *A. campestris. A* aff. *basii* was considered important because it was frequently used, had a good taste, and was valued by the locals. *A. campestris* was also detected in great abundance, valued and eaten traditionally, and thought to have health benefits. *Ramaria* and *Russula* mushrooms were also considered important; individuals referred to these mushrooms more often than others and noted that these mushrooms could be cooked on their own without having to add meat.

The diversity of mushroom species in the *Russula* genus used by the SMH community and the variety of *Amanita* and *Ramaria* species observed was incredible. Based on these findings, the CS of these mushrooms to the SMH community reflects their value as multifunctional foods.

The present study also showed that habitats outside the forest are important and have useful resources. Mushrooms growing in these areas, such as *A. campestris*, *C. cyathiformis*, and *M. oreades*, were all used by the locals.

The EMCSI used in Oaxaca was successfully modified using SMH-specific characteristics and was useful for determining the CS of mushrooms in SMH, Tlaxcala.

## Abbreviations

CS: Cultural significance; FM: Frequency of mention; OM: Order of mention; SMH: San Mateo Huexoyucan; EMCSI: Index of cultural significance for wild edible mushrooms; EMCSPI: Edible mushroom cultural significance pondered index; PAI: Perceived abundance index; FUI: Frequency of use index; TSAI: Taste score appreciation index; MFFI: Multifunctional food index; KTI: Knowledge transmission index; HI: Health index; EMCSPI: Edible mushroom cultural significance pondered index; MI: Mention index; ORV: Ordinal rank value; PCA: Principal components analysis; MDS: Multi-dimensional scaling.

## Competing interests

The authors declare that they have no competing interests.

## Authors’ contributions

MA and AALE–Designed sampling scheme, collected mushrooms, drafted the manuscript, and performed the ordination and data analyses. AK–Identified the mushrooms and interpreted the data. RGO and AET–Participated in the data analysis and mushroom collection. All authors read and approved the final manuscript.
